# Profound Sensorineural Hearing Loss Following Typhoid Fever in a Young Adult Female: A Rare Complication

**DOI:** 10.1155/crdi/5514866

**Published:** 2025-12-22

**Authors:** Nischal Shrestha, Priti Khanal, Gyan Raj Aryal, Aasha Shahi

**Affiliations:** ^1^ Department of Internal Medicine, Nobel Medical College, Biratnagar, Nepal; ^2^ Department of Otorhinolaryngology, Nobel Medical College, Biratnagar, Nepal; ^3^ Department of Internal Medicine, Kathmandu Medical College, Kathmandu, Nepal, kmc.edu.np

**Keywords:** cochlear implants, sensorineural hearing loss, typhoid fever

## Abstract

Typhoid fever, caused by Salmonella Typhi and Salmonella Paratyphi A, remains endemic in Nepal. Typhoid fever can affect nearly all systems of the body, leading to various complications; however, sensorineural hearing loss (SNHL) remains an exceptionally rare occurrence. This case report describes a 24‐year‐old female school teacher who presented with an 8‐day history of fever, followed by a 6‐day history of bilateral hearing loss and tinnitus. She was diagnosed with typhoid fever based on a positive Widal test and was treated with intravenous antibiotics, antipyretics, and oral steroids, but her hearing did not improve. She was referred to a tertiary center for further evaluation, where she was diagnosed with profound SNHL. A cochlear implant was recommended but declined by the patient due to financial constraints and uncertainty regarding the outcome. This case highlights the need for further research into the pathogenesis and treatment of SNHL associated with typhoid fever.

**Key Clinical Message**

• Sensorineural hearing loss (SNHL) is a rare but significant complication of typhoid fever. While the addition of steroids to the treatment regimen may reduce morbidity, more research is needed as outcomes remain variable.

## 1. Introduction

Salmonella enterica serovars Typhi and Paratyphi A are human‐restricted pathogens responsible for typhoid and paratyphoid fever, collectively known as enteric fever [1]. The World Health Organization (WHO) estimates an annual typhoid prevalence of 0.3% in Nepal [2]. Typhoid fever can affect multiple organ systems, leading to various complications, including sensorineural hearing loss (SNHL), though this is rare [3, 4]. This report presents a young adult female with profound SNHL following typhoid fever, highlighting an unusual and uncommon clinical course.

## 2. Case Presentation

### 2.1. Case History and Examination

A 24‐year‐old female school teacher presented to the outpatient clinic with an 8‐day history of fever accompanied by chills and a 6‐day history of bilateral hearing loss and tinnitus. The hearing loss was sudden in onset and nonfluctuating. The tinnitus was high‐pitched and continuous. There were no episodes of vertigo, no preceding upper respiratory tract infection, and no nasal obstruction or discharge. She had been self‐medicating with paracetamol but had not taken any other medications. There was no history of trauma, ototoxic drug exposure, or any other systemic illnesses.

On examination, she was febrile with a temperature of 38.7°C, a pulse of 94 beats/min, a respiratory rate of 22 breaths/min, and blood pressure of 110/60 mmHg. Her hearing was severely impaired hearing bilaterally. Otoscopic examination showed intact tympanic membranes, and no discharge was observed. Nasal examination was unremarkable, with healthy mucosa and no evidence of congestion or sinus tenderness. Other systemic findings were unremarkable.

### 2.2. Diagnosis, Investigation, and Treatment

Laboratory evaluation revealed mild leukocytosis and elevated C‐reactive protein (CRP) at 65 mg/L (normal < 10 mg/L). Liver function tests were within normal limits. Serologic tests for dengue, malaria, and leptospirosis were negative. A positive Widal test (O titer ≥ 1:160), in conjunction with clinical features, confirmed the diagnosis of typhoid fever. Pure tone audiometry demonstrated profound bilateral SNHL (Figure 1).

Figure 1Audiogram showing that both air conduction and bone conduction thresholds are above 80 dB across all tested frequencies, with an air–bone gap of less than 10 dB, confirming profound bilateral sensorineural hearing loss (SNHL). (a) Pretreatment: profound bilateral SNHL noted; no spontaneous recovery. (b) Posttreatment (1 month after steroids): hearing loss persisted with no measurable improvement.

**Figure:**
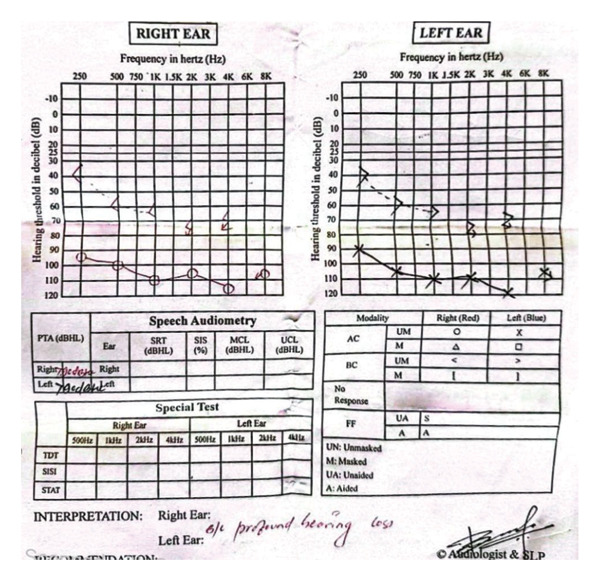
(a)

**Figure:**
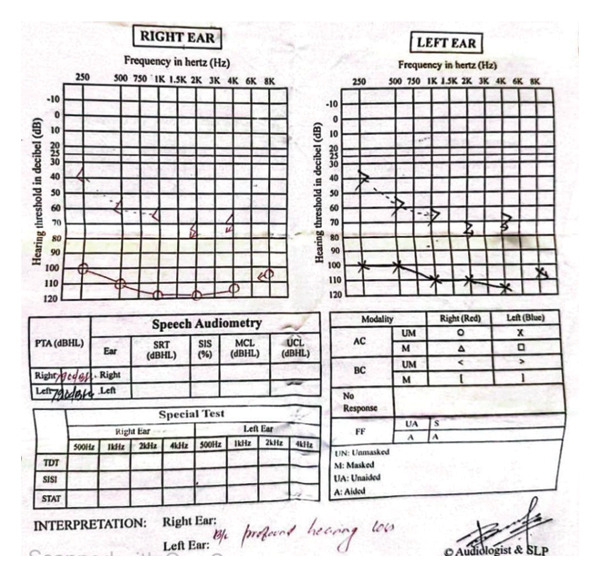
(b)

There was no history of prior ear surgery or tympanoplasty, and no baseline audiometry was available before the onset of hearing loss. She was started on intravenous ceftriaxone (2 g every 12 h), oral paracetamol, and prednisolone at 1 mg/kg/day, tapered over time.

### 2.3. Outcome and Follow‐Up

Although her fever resolved, there was no improvement in her hearing despite a short course of corticosteroid therapy. She was referred immediately to a tertiary center for further evaluation and management, where repeat audiometry reconfirmed profound bilateral SNHL. At the higher center, she was followed up intermittently, but her hearing deficit persisted without improvement. According to follow‐up information, hearing aids were trialed without success, and she was eventually advised to undergo cochlear implantation, which she declined due to financial constraints and uncertainty regarding the outcome. After 1 year, she returned to our hospital for disability certification due to permanent hearing loss.

## 3. Discussion

The classic symptoms of typhoid fever usually develop gradually, beginning with fever, chills, and fatigue, followed by abdominal discomfort, nausea, and either constipation or diarrhea in the second week [4]. However, the clinical spectrum of typhoid fever has broadened, particularly in tropical and endemic regions, where atypical presentations are increasingly reported [5]. In our patient, for example, the disease presented unusually with fever, bilateral hearing loss, and tinnitus. The Widal test is often used as the primary diagnostic tool for typhoid in developing countries due to cost‐effectiveness and ease of use, despite the availability of more accurate methods such as blood or stool cultures, which require specialized training [6].

A detailed physical examination, including otoscopy, is usually performed to assess the ear, as was done in this case. The Weber and Rinne tuning fork tests, combined with pure tone audiometry, can differentiate between conductive and SNHL [7].

While SNHL is associated with viral rather than bacterial infections [3], in this case, the hearing loss was attributed to Salmonella Typhi, the bacterium responsible for typhoid fever. There is no established population‐based incidence of SNHL following typhoid fever, as it has only been documented in rare case reports and small case series. To date, fewer than a dozen cases have been reported in the literature, highlighting its rarity. For example, Escajadillo et al. reported six cases of cochleovestibular lesions secondary to typhoid, but larger studies are lacking. This makes it difficult to determine the true incidence or risk factors for hearing loss in affected individuals [3, 4, 8].

A previous study of six cases with cochleovestibular lesions from typhoid fever found that SNHL typically developed between the second and third weeks of the illness, with a predilection for the left ear. Follow‐up audiometric testing was available in four of these patients: hearing worsened in one, improved in two, and remained unchanged in one [6]. In our patient, however, SNHL was bilateral. The pathophysiology behind SNHL in typhoid fever remains unclear; however, it is hypothesized that endotoxins produced by Salmonella Typhi may damage the organ of Corti, cause capillary congestion, and result in cochlear hydrops [8]. Recovery from SNHL is variable, with spontaneous improvement in 32%–65% of the cases. Prognosis depends on factors such as age, the degree of hearing loss, the audiometric pattern, and the timeliness of treatment [7].

In endemic areas, typhoid fever is generally treated with oral antibiotics. Fluoroquinolones, such as ciprofloxacin and ofloxacin, are often used for multidrug‐resistant cases [9]. In this case, our patient was treated with IV ceftriaxone. There are limited data regarding the use of systemic steroid in patients with typhoid fever complicated by SNHL. A randomized trial conducted in Indonesia found that adding dexamethasone to chloramphenicol therapy reduced mortality rates without increasing complications. The precise mechanism by which steroids may improve SNHL is not fully understood; however, it is thought that they help reduce inflammation and swelling within the cochlea [4]. Further research is needed to clarify the efficacy of steroids in managing SNHL following typhoid fever.

Currently, no limited data exist for SNHL in typhoid fever, due to the rarity of the condition. However, the timely intervention is critical, as studies suggest that delays in treatment and a downsloping audiometric curve are associated with worse recovery outcomes [10]. Glucocorticoids are typically recommended early in the course of SNHL, with a daily 30–60 mg of oral prednisone, tapered over 1‐2 weeks [11]. In this case, the patient was started on 1 mg/kg/day of oral prednisolone in a tapering dose. However, there was no improvement in her hearing, underscoring the challenges in managing such rare complications.

Given the lack of response to medical treatment, cochlear implantation was suggested as the next step. Cochlear implants are effective for treating profound hearing loss, particularly when speech perception is poor bilaterally. While outcomes following cochlear implantation are generally favorable, they can vary, with the duration of deafness being a key predictor of success [12]. However, our patient declined the implant due to financial concerns. This case illustrates the challenges faced in low‐resource settings, where financial barriers may limit access to advanced treatments like cochlear implantation.

Preventive strategies remain essential in reducing the incidence of typhoid fever and its complications. Public health measures such as improved sanitation, safe drinking water, and widespread typhoid vaccination are crucial in endemic regions [13]. Clinicians should be alert to atypical presentations, including early auditory symptoms like tinnitus or hearing difficulty during febrile illness. Prompt diagnosis and initiation of appropriate antibiotics, guided by local resistance patterns, are the key to preventing systemic and neurological sequelae. When SNHL develops, corticosteroid therapy may be considered to reduce inflammation, although evidence supporting its efficacy remains limited. For patients with irreversible profound hearing loss, timely referral for auditory rehabilitation, including cochlear implantation, should be encouraged where resources permit.

## 4. Conclusion

This case highlights profound bilateral SNHL as a rare but disabling complication of typhoid fever. Despite the patient’s young age and timely treatment with corticosteroids, her hearing did not improve. The case emphasizes the need for heightened clinical awareness and further research into effective management strategies for SNHL associated with typhoid fever.

## Ethics Statement

Written informed consent was obtained from the patient to publish this report in accordance with the journal’s patient consent policy.

## Disclosure

A preprint version of this manuscript was previously made available on Authorea (https://www.authorea.com/users/514825/articles/1222241). The present version includes revisions following peer review, with updated authorship.

## Conflicts of Interest

The authors declare no conflicts of interest.

## Author Contributions

Nischal Shrestha: conceptualization, data curation, investigation, methodology, project administration, resources, software, writing–original draft, and writing–review and editing. Priti Khanal: original draft and writing–review and editing. Gyan Raj Aryal: supervision, investigation, and writing–review and editing. Aasha Shahi: writing–review and editing.

## Funding

No funding was received for this research.

## Data Availability

All data supporting the findings of this case report are included within the article. Further data will be provided upon request.
